# Supplementary material for: Van der Waals epitaxial growth and optoelectronics of large-scale WSe2/SnS2 vertical bilayer p–n junctions
Source: Nat Commun. 2017 Dec 4;8:1906. doi: 10.1038/s41467-017-02093-z (PMC5715014; doi:10.1038/s41467-017-02093-z)
Supplement: Supplementary file 1 — Supplementary Information [file 41467_2017_2093_MOESM1_ESM.pdf]

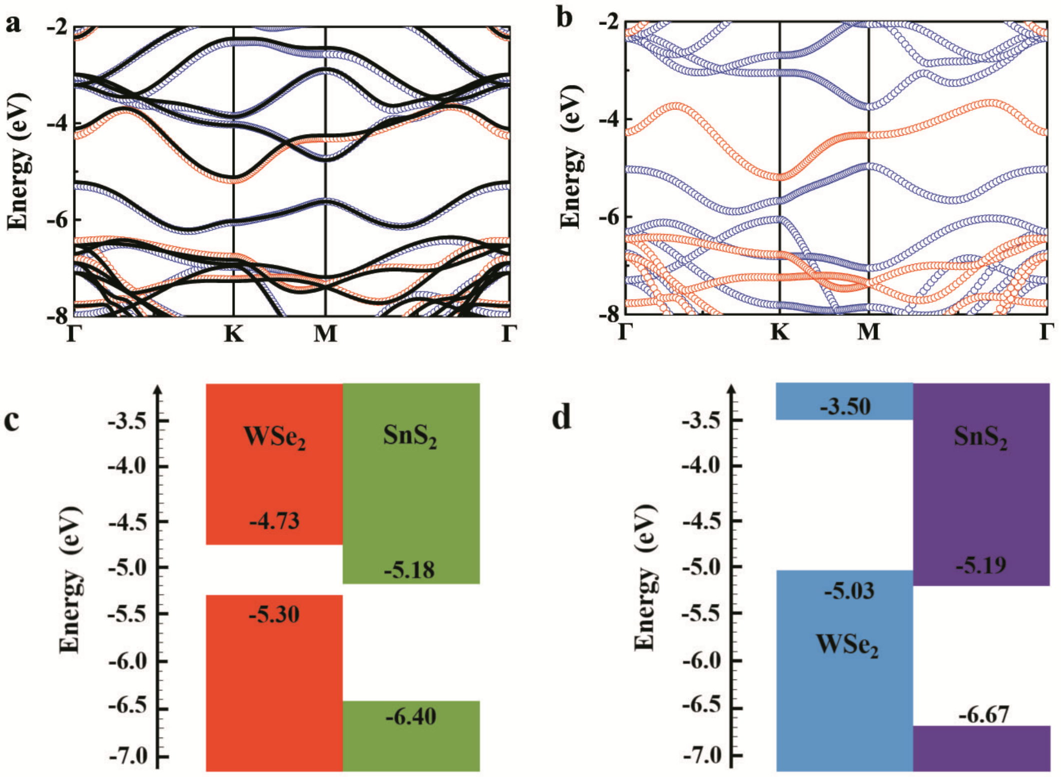

Supplementary Figure 1. Electronic band structures of the WSe_2/SnS_2 heterojunction.

(a) with the same lattice constants for the single-layer WSe_2 and SnS_2 layers, and **(b)** with different lattice constants for WSe_2 and SnS_2 layers. In (a), the black lines represent the band structure of the WSe_2/SnS_2 heterojunction, directly from the DFT calculation for the bilayer model. The blue and red dots represent the band structure of the free-standing single-layer WSe_2 and SnS_2, both with the same lattice constants as that in the bilayer model. In (b), the band structure of heterojunction is obtained by combining the separate results of free-standing single-layer WSe_2 and SnS_2 layers, with their simulation cells fully relaxed. The corresponding band alignments of the WSe_2/SnS_2 heterojunction **(c)** with the same lattice constants for single-layer WSe_2 and SnS_2, and **(d)** with different lattice constants for WSe_2 and SnS_2 layers. The vacuum level is set as 0 eV.

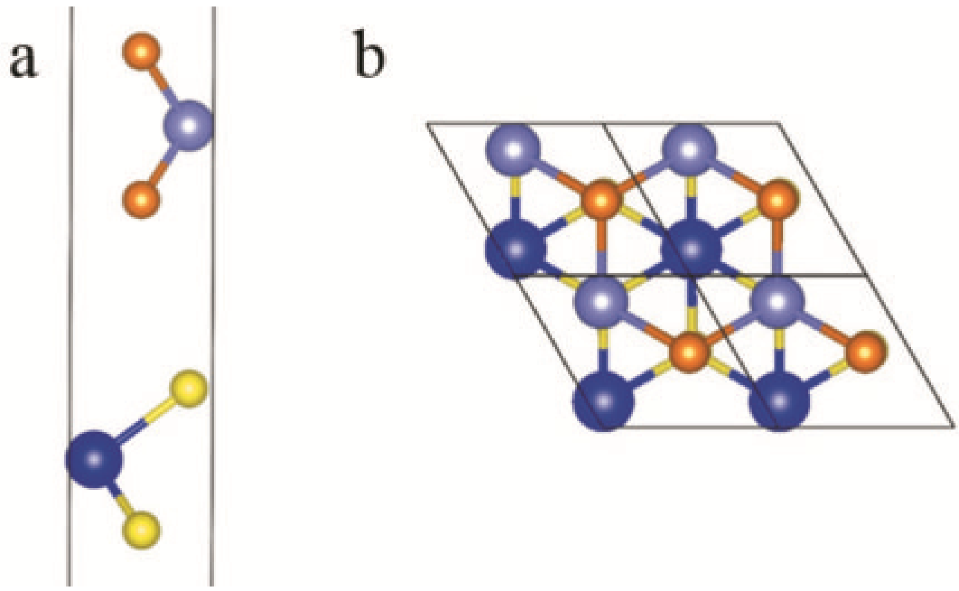

Supplementary Figure 2. Atomic structures of high-symmetry stacking orders of WSe_2/SnS_2 heterojuction with side (a) and top (b) views.

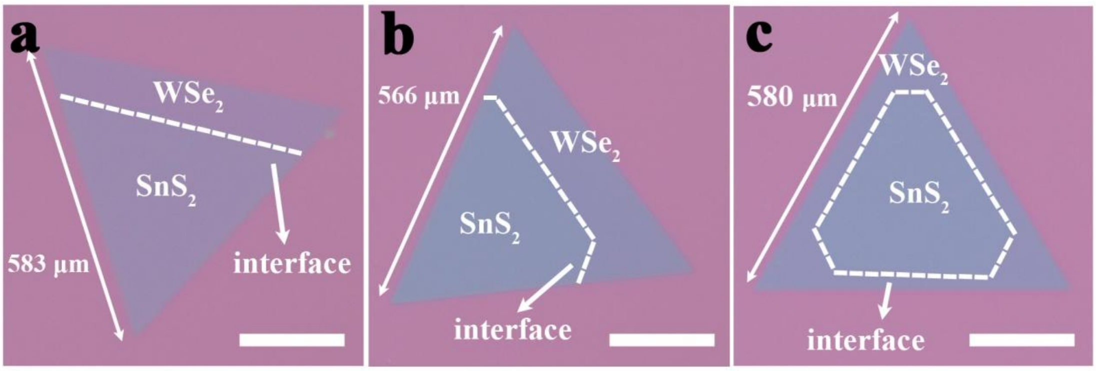

Supplementary Figure 3. Typical optical images of as-grown WSe_2/SnS_2 vertical heterostructures.

(a) Triangle shape SnS_2 covered on the corner of bottom WSe_2 layer. (**b)** Irregular shape SnS_2 covered on the corner of bottom WSe_2 layer. (**c)** Hexagon shape SnS_2 covered on the center of bottom WSe_2 layer. All the scale bars: 100 µm.

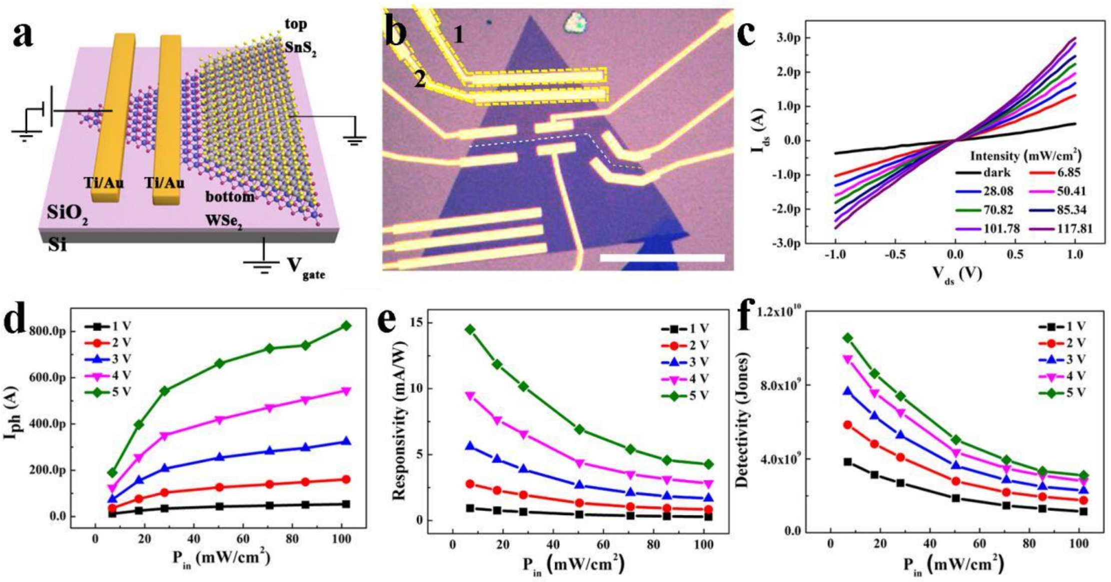

Supplementary Figure 4. Photoresponse characterization of pristine WSe_2 device.

(a) A cartoon schematic of the pristine WSe_2 photodetector. (**b)** Optical image of the pristine WSe_2 device. (measured using electrodes 1 and 2). (**c)** I_{ds}-V_{ds} curves under 520 nm laser illumination at different incident power. V_{ds} range from -1 V to 1 V. **(d)** Dependence of photocurrent on illumination power intensities at different bias voltage. (**e)** Photoresponsivity of the pristine WSe_2 photodetector under various illumination power intensities. (**f)** Photodetectivity of the pristine WSe_2 photodetector under various illumination power intensities.

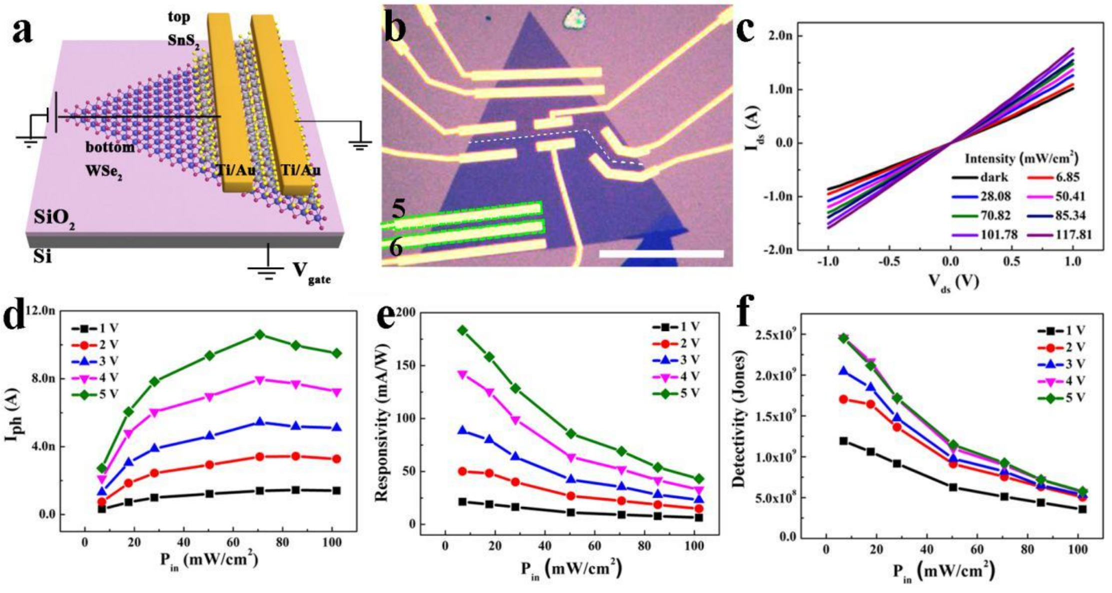

Supplementary Figure 5. Photoresponse characterization of the parallel mode WSe_2/SnS_2 heterostructure device.

(a) A cartoon schematic of the parallel mode WSe_2/SnS_2 heterostructure photodetector. (**b**) Optical image of the parallel mode WSe_2/SnS_2 heterostructure photodetector. (measured using electrodes 5 and 6). (**c**) I_{ds}-V_{ds} curves under 520 nm laser illumination at different incident power. V_{ds} range from -1 V to 1 V. **(d)** Dependence of photocurrent on illumination power intensities at different bias voltage. (**e**) Photoresponsivity of the parallel mode WSe_2/SnS_2 heterostructure photodetector under various illumination power intensities. (**f**) Photodetectivity of the parallel mode WSe_2/SnS_2 heterostructure photodetector under various illumination power intensities.

Supplementary Notes

Supplementary Note 1.

Our electronic band structure calculations were performed based on the density functional theory (DFT), as implemented in the Vienna ab initio package (VASP).[1,2] We use the projector-augmented wave (PAW) methods and the generalized gradient approximation (GGA)[3] for the exchange–correlation functional. The optB88-vdW[4] functional was chosen so as to obtain a good description of the dispersion forces. The cutoff energy was 450 eV. All the atoms in the slab were allowed to fully relax until the forces acting on them are less than 0.01 eV $Å^{-1}$. A slab layer of 25 Å thick was enough to avoid interactions between the layers. The Monkhorst–Pack (M-P) scheme was used to sample the Brillouin zone, and the k-mesh was set as 15 × 15 × 1 for the geometry optimization and the static calculations.

Firstly, the electronic band structure of WSe_2/SnS_2 heterostructure was calculated by using the bilayer model as shown in Fig. 2. The results are shown in Fig. 1 (a) and (c). After fully relaxing the simulation cell, the lattice spacings of WSe_2 and SnS_2 layers are 0.31 nm. We also calculate the band structures of free-standing WSe_2 and SnS_2 layers, with their lattice constants fixed as that in this bilayer model. The results are also shown in Fig. 1 (a) as blue and red dots. We can find that, with the formation of heterostructure, the van der waals interactions between layers will not affect the band structures of free-standing WSe_2 and SnS_2 layers severely. In our experiment, The SAED pattern shows two sets of electron diffraction patterns, corresponding to WSe_2 and SnS_2, respectively, which means the WSe_2 and SnS_2 possess different lattice spacings. In order to include the influence of the contrasting lattice cells on the band alignment, we separately calculate the band structures of free-standing single-layer WSe_2 and SnS_2. The lattice spacings are 0.28 nm for WSe_2 and 0.33 nm for SnS_2, in agreement with our experimental results well. Considering the fact that the dispersion force only slightly changes the band structures of WSe_2 and SnS_2 layers, we combine the results for two separate layers and show them in Figs. 1 (b) and (d), as the band structure and the band alignment of WSe_2/SnS_2 heterojuction.

Supplementary Note 2.

The blue, yellow, purple and orange atoms represent the Sn, S, W and Se atoms, respectively.

The solid lines demarcate the unit cell.

Reference

1 G. Kresse, J. F. Efficient iterative schemes for ab initio total-energy calculations using a plane-wave basis set. *Physical Review B* **54**, 11169-11186 (1996).

2 G. Kresse, D. J. From ultrasoft pseudopotentials to the projector augmented-wave method. *Physical Review B* **59**, 1758-1775 (1999).

3 John P. Perdew, K. B., Matthias Ernzerhof. Generalized Gradient Approximation Made Simple. *Physical review letters* **77**, 3865-3868 (1996).

4 Klimeš, J., Bowler, D. R. & Michaelides, A. Van der Waals density functionals applied to solids. *Physical Review B* **83**, 1-13 (2011).
